# Effect of Hydrophobic Chain Length in Amphiphilic Chitosan Conjugates on Intracellular Drug Delivery and Smart Drug Release of Redox-Responsive Micelle

**DOI:** 10.3390/md22010018

**Published:** 2023-12-27

**Authors:** Yuting Yuan, Wenqiang Tan, Yingqi Mi, Linqing Wang, Zhen Qi, Zhanyong Guo

**Affiliations:** 1 Key Laboratory of Coastal Biology and Bioresource Utilization, Yantai Institute of Coastal Zone Research, Chinese Academy of Sciences, Yantai 264003, China; ytyuan@yic.ac.cn (Y.Y.); yqmi@yic.ac.cn (Y.M.); linqingwang@yic.ac.cn (L.W.); 2 University of Chinese Academy of Sciences, Beijing 100049, China; 3 Center for Ocean Mega-Science, Chinese Academy of Sciences, Qingdao 266071, China; 4 College of Life Sciences, Yantai University, Yantai 264005, China; qzqq2022@163.com

**Keywords:** different hydrophobic chain length, low molecular weight chitosan, trigger-release

## Abstract

Three redox-sensitive nanocarriers were rationally designed based on amphiphilic low molecular weight chitosan-cystamine-octylamine/dodecylamin/cetylamine (LC-Cys-OA, LC-Cys-DA, LC-Cys-CA) conjugates containing disulfide linkage for maximizing therapeutic effect by regulating hydrophobic interaction. The resultant spherical micelles had the characteristics of low CMC, suitable size, excellent biosafety and desired stability. The drug-loaded micelles were fabricated by embedding doxorubicin (Dox) into the hydrophobic cores. The effect of hydrophobic chain lengths of amphiphilic conjugates on encapsulation capacity, redox sensitivity, trigger-release behavior, cellular uptake efficacy, antitumor effect and antimigratory activity of Dox-loaded micelles was systematically investigated. Studies found that Dox-loaded LC-Cys-CA micelle had superior loading capacity and enhanced redox sensitivity compared with the other two micelles. Release assay indicated that the three Dox-loaded micelles maintained sufficiently stability in normal blood circulation but rapidly disintegrated in tumor cells. More importantly, the LC-Cys-CA micelle with a longer hydrophobic chain length exhibited a higher accumulative Dox release percentage than the other two micelles. Additionally, an increase in hydrophobic chain lengths of amphiphilic conjugates improved cellular uptake efficiency, antitumor effect and antimigration activity of Dox-loaded micelles, which could be explained by enhanced loading ability and redox sensitivity. Our research was expected to provide a viable platform for achieving a desired therapeutic efficacy via the alteration of hydrophobic interaction.

## 1. Introduction

Facing the unrestrained growth and proliferation of cancer cells, traditional chemotherapy drugs with various limitations (poor water solubility, rapid blood clearance, low target and severe side effects) could not meet the high demand for current cancer therapy [1]. It was urgent to develop an intelligent drug delivery system to improve therapeutic effects. Among many nanocarriers, polymeric micelles stood out owing to various exceptional traits like extending blood circulation time and improved tumor cell accumulation by the enhanced permeability and retention (EPR) effect [2]. However, recent research demonstrated that polymeric micelles as drug carriers were not only capable of delivering an encapsulated drug deeply into target tissues but also needed to quickly accomplish drug release to efficiently kill various cancer cells. Therefore, many researchers have shifted their focus to responsive polymer micelles. Responsive polymer micelles have a wide range of applications (including drug delivery systems, biosensors, actuators, tissue engineering scaffolds) and have attracted tremendous attention. Compared with conventional polymers micelles, responsive polymer micelles have many outstanding advantages, such as tailored and tunable properties, enhanced functionality, response to dynamic environments, triggered response and on-demand behavior [3]. Among many applications, responsive polymer micelles used as drug delivery carriers were extremely important in the field of biomedicine, as they could respond to dynamic environments and improve drug release efficiency from polymeric micelles and further largely avoid drug resistance caused by repeated use of large doses of cancer drugs.

The tumor microenvironment-responsive micelles could rapid disassemble and subsequently trigger burst release of encapsulated drugs in the specific tumor tissue while maintaining the common advantages of polymer micelles. The development of redox-sensitive polymeric micelles was of particular importance among various stimuli-sensitive micelles. To the best of our knowledge, glutathione (GSH) was believed to mainly exist in the cytoplasm (2–10 mM) but not in the plasma (2–20 μM), and an obviously remarkably increased GSH concentration (4–5 times) was observed in tumor cells compared with normal cells, which was the prerequisite for designing a desired redox-sensitive polymer micelle [4]. These redox-sensitive micelles with a characteristic disulfide bond had the ability to maintain an intact structure in normal physiological conditions and were conducive to inhibit undesirable inactivation and degradation of encapsulated drugs before reaching tumor cells. Once entering the target tissue, the disulfide bonds of redox-sensitive micelles were prone to be destroyed rapidly by redox reaction, resulting in micellar decomposition followed by achieving tumor-specific drug burst release. Meanwhile, the position of the disulfide bond in micelle had a great influence on the degradation rate and antitumor activity of drug-loaded micelles. Liu’s group reported that the disulfide bond located in the junction of the hydrophilic and hydrophobic group had superior sensitive to stimuli-environment than that positioned in the center core [5].

Considering the useful practicality of redox-responsive micelles, low molecular weight chitosan (LC) has become a focus of attention and is widely used to design advanced biomaterials for application in biomedical fields; this might be attributed to its superior characteristics such as good water solubility, wonderful bio-safety, prominent biodegradability and easy modification of structure [6]. Lin et al. prepared novel low molecular weight chitosan/lignosulfonate (CS-LS) micelle by the polyelectrolyte self-assembly method. The result displayed that CS-LS micelle had a particle size of 239 nm and improved the stability and antioxidant activity of encapsulated bioactive substance [7]. Itoo et al. designed low molecular weight chitosan/vitamin E (Ch/VES) micelle by two steps and further self-assembled into core-shell structural micelle. The drug @ Ch/VES micelle displayed a particle size of 282.7 nm and loading content (DLC) of 5.28% when the drug was loaded into the micelle by the ratio of 1:4. Meanwhile, the drug @ Ch/VES micelle could significantly inhibit the growth of tumor cells and decrease nephrotoxicity [8]. It was reported that micelles were internalized into cells through the endocytosis pathway, which was relevant to the surface characteristics of micelles (such as particle size, zeta potential and shape) and cell type. For instance, nanocarriers with a particle size larger than 300 nm were easier to be cleared by the reticuloendothelial system (RES). In contrast, a nanocarrier with a diameter of less than 300 nm was favorable for delivery into tumor cells by clathrin-mediated endocytosis [9]. Therefore, a deeper understanding of the influence factors of cellular uptake of nanocarriers could help the design of better drug delivery systems for application in cancer therapy. Liu et al. prepared three self-assembled micelles based on chondroitin sulfate-deoxycholic acid conjugates (CSAD) with a different degree of substitution (DS) of the hydrophobic group. A smaller size, higher loading efficiency, enhanced accumulative drug release and increased cytotoxicity against MCF-7 cells were observed with the DS of hydrophobic group increased [10]. Theoretically, the effects of different chain lengths of hydrophobic groups on the physicochemical properties of nanocarriers might be similar to DS [11]. Hence, we hypothesized that the optimized physicochemical properties, enhanced intracellular uptake efficiency, increased antitumor effect and improved antimigration activity of redox-responsive micelles were achieved by regulating the chain length of hydrophobic segments.

For all of the above reasons, a series of novel redox-sensitive micelles with different hydrophobic chain lengths based on amphiphilic low molecular weight chitosan-cystamine-octylamine/dodecylamine/cetylamine conjugates (LC-Cys-OA, LC-Cys-DA and LC-Cys-CA) were fabricated by combination of the EPR effect and redox-responsive behavior to obtain high therapeutic efficacy via changing hydrophobic interactions. LC served as a hydrophilic segment due to good water solubility, excellent biodegradability and biosecurity. Meanwhile, OA, DA and CA were regarded as hydrophilic segments to provide different hydrophobic forces. The redox-sensitive behaviors of micelles could be realized by disulfide bonds, which are located at the junction of the hydrophilic and hydrophobic segments. The hydrophobic Dox was wrapped into the core of the micelle in the process of self-assembly. Subsequently, three Dox-loaded micelles specifically internalized into tumor tissues by the EPR effect and the encapsulated Dox was released quickly in high GSH concentration of tumor cells to inhibit the growth of tumor cells. The chemical structure of three amphiphilic conjugates (LC-Cys-OA, LC-Cys-DA and LC-Cys-CA) were confirmed by FTIR and ^1^H NMR spectroscopy. The physicochemical properties of blank micelles and Dox-loaded micelles were systematically evaluated by fluorescence spectroscopy, transmission electron microscopy (TEM), dynamic light scattering (DLS) and UV–vis spectroscopy. The redox sensitivity of the three micelles were verified by observing the change of particle size distribution and determining the Dox release behavior in different media. The intracellular uptake efficiency, antitumor effect and antimigration activity of the three Dox-loaded micelles were studied by combining confocal laser scanning microscopy (CLSM), flow cytometry (FCM), Thiazolyl Blue Tetrazolium Bromide (MTT) method and scratch assay. Moreover, the effect of the hydrophobic chain lengths of the three redox-sensitive micelles on the above properties was mainly investigated. In this work, Doxorubicin as a hydrophobic chemotherapy drug was encapsulated in the hydrophobic core of the three prepared micelles. After Dox-loaded micelles entered the tumor cells, Dox was released from micelles in response to high concentration glutathione. Hence, the three prepared Dox-loaded micelles can be used in the treatment of various cancers (such as lung cancer, breast cancer, colorectal cancer, ovarian cancer, neuroblastoma, rhabdomyosarcoma and so on).

## 2. Results and Discussion

### 2.1. Synthesis and Structural Characterization of Amphiphilic LC-Cys-OA/LC-Cys-DA/LC-Cys-CA Conjugates

**Figure 1:**
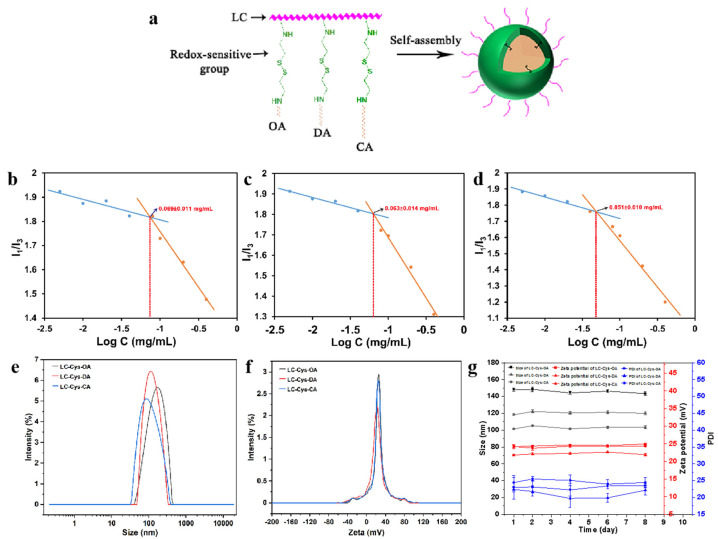
Schematic illustration of the formation process of blank micelles with different hydrophobic chain lengths (**a**). CMC value (**b**–**d**), particle size (**e**), zeta potential (**f**) and storage stability (**g**) of LC-Cys-OA, LC-Cys-DA and LC-Cys-CA micelles.

To achieve a smart cancer therapy with highly efficient delivery and intelligent release in the tumor environment, three novel amphiphilic conjugates with a redox-sensitive property were designed and prepared [12]. The detailed synthesis process is illustrated in Figure S1a. The amino groups and hydroxy groups of LC were activated by the catalytic action of CDI and continued to react with Cys·2HCl. To obtain the LC-Cys conjugate in Figure 1a, we have controlled strictly the feeding ratio of LC and Cys·2HCl (1:2) and the order and speed of drop in this experiment. Subsequently, the primary product (LC-Cys) was further reacted with the amino group of OA, DA and CA via acylation reaction to obtain three redox-sensitive amphiphilic conjugates.

The chemical structures of LC and three amphiphilic conjugates were confirmed by FT-IR and ^1^H NMR. As depicted in Figure S1b,c, the representative stretching vibrations of N-H and O-H of LC were found at 3384 cm^−1^. The absorption peak of 2923 cm^−1^ was caused by C–H stretching vibration of CH_3_. The N–H bending vibration of NH_2_ was 1631 cm^−1^ and the peak at 1069 cm^−1^ might belong to the C–O–C stretching vibration of LC [13]. After Cys·2HCl grafting to LC, the new peak, appearing at 1752 cm^−1^, 1708 cm^−1^ and 1661 cm^−1^, might be caused by the C=O stretching vibration band due to the formation of imidazole methyl, estercarbamic acid ester and urea linkages [14]. In the three amphiphilic conjugates, the absorption peak around 2923 cm^−1^ and 2853 cm^−1^ obviously strengthened, which might be caused by the C–H stretching vibration of CH_2_ or CH_3_. It was excellent data for successful synthesis of LC-Cys-OA, LC-Cys-DA and LC-Cys-CA.

In ^1^H NMR spectra (Figure S1c), the chemical shift at 2.97 ppm and 3.45–4.03 ppm was the signals of H_2_ and H_3–6_ of LC. The typical peaks of LC-Cys appeared at 3.25 ppm and 2.60–2.87 ppm, which were consistent with previous reports [15]. The new signals at 0.83–1.22 ppm in the spectra of the LC-Cys-OA, LC-Cys-DA and LC-Cys-CA conjugates confirmed the successful grafting of OA, DA and CA to the LC-Cys, which belonged to the characteristic peaks of methylene and methine groups. Based on the location and the peak intensity of the hydrogen signal of OA, DA and CA in the three amphiphilic conjugates, the DS of LC-Cys-OA, LC-Cys-DA and LC-Cys-CA conjugates were 60%, 58% and 54%, respectively.

### 2.2. Preparation and Characterization of LC-Cys-OA/LC-Cys-DA/LC-Cys-CA Micelles

The amphiphilic conjugates based on LC-Cys-OA, LC-Cys-DA and LC-Cys-CA could self-assemble into core–shell micelles by an ultrasound-dialysis method. The self-assembly behavior is shown in Figure 1a. OA, DA and CA were used as hydrophobic groups and formed the core of the micelles, while LC with good water solubility regarded as the hydrophilic group formed the shell of the micelle. Meanwhile, cystamine with a redox-response property acted as a bridge that connected the hydrophilic groups and hydrophobic groups.

The CMC values of three blank micelles were studied using hydrophobic pyrene [16,17,18]. Figure 1b–d shows that the CMC values of the three blank micelles were 0.069 ± 0.011 mg/mL, 0.063 ± 0.014 mg/mL and 0.051 ± 0.010 mg/mL, respectively. Subsequently, a TEM instrument was applied to observe the micro-morphology of the three prepared blank micelles. As demonstrated in Figure S2, a spherical nano-micelle was presented in a TEM image. Next, the DLS result showed that the average particle sizes of LC-Cys-OA, LC-Cys-DA and LC-Cys-CA micelles were 147.95 nm, 118.24 nm and 101.47 nm, respectively (Figure 1e). The zeta potential of the three blank micelles was a positive value (Figure 1f). With the length of hydrophobic groups increased, the average particle sizes of the three blank micelles tended to decrease, but the zeta potential of the three blank micelles remained basically unchanged. In addition, the particle sizes, zeta potential and PDI of the three micelles had no obvious change within a period of 8 days (Figure 1g), which provided excellent evidence about the storage stability of the three blank micelles.

### 2.3. Biosafety Evaluation

**Figure 2:**
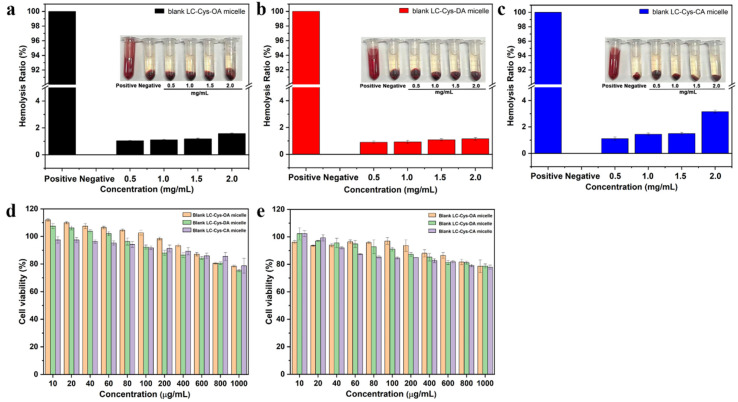
The hemolysis assay of blank micelles with different hydrophobic chain lengths (**a**–**c**). Cell viability of the L929 cell (**d**) and A549 cell (**e**) after treatment with the three blank micelles for 48 h.

As displayed in Figure 2a–c, the hemolysis ratios of the three redox-sensitive micelles with various concentrations from 0.5–2.0 mg/mL were lower than 5%, indicating that these three micelles had good blood compatibility and were suitable for intravenous injection. Furthermore, the cell viability of the three blank micelles against L929 cells and A549 cells was detected. As illustrated in Figure 2d,e, when the concentration increased to 1000 μg/mL, the three micelles exhibited a weak inhibitory effect on L929 cells and A549 cells. The cell viabilities of the three prepared micelles were still above 75% for 48 h incubation. Based on all the above data, it was proved that the three prepared micelles could be excellent materials for efficient intracellular drug delivery [19].

### 2.4. Redox-Triggered Disassembly Behavior of Three Blank Micelles

**Figure 3:**
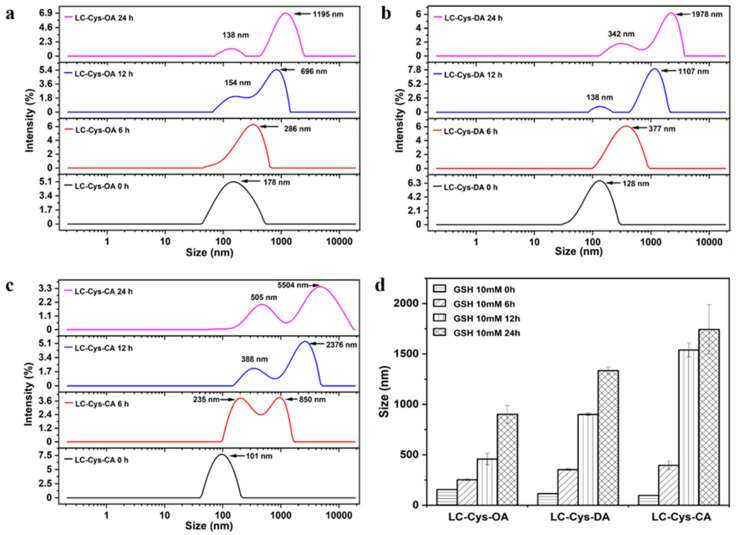
Change of size distribution of the LC-Cys-OA (**a**), LC-Cys-DA (**b**) and LC-Cys-CA (**c**) micelles in the presence of 10 mM GSH. Change of particle size of the three prepared micelles dealt with 10 mM GSH (**d**).

The redox-responsive behaviors of the three prepared blank micelles were evaluated by observing the changes of particle size and size distribution within 24 h under a 10 mM GSH condition [20]. In a view of Figure 3, the three blank micelles exhibited a narrow size distribution without GSH incubation. A bimodal distribution appeared as three micelles treated with GSH for 12 h. Meanwhile, LC-Cys-OA, LC-Cys-DA and LC-Cys-CA exhibited obvious changes in particle size, which increased from 156.31 nm to 902.21 nm, 114.94 nm to 1334.63 nm and 97.87 nm to 1742.73 nm over 24 h, respectively. It was proved that these three micelles were sensitive to the redox environment of cancer cells. Moreover, the result indicated that the redox-sensitive disassembly behavior of the three blank micelles was closely associated with the hydrophobic chain length of micelles. The LC-Cys-CA micelle had an obviously increased size compared with the LC-Cys-OA and LC-Cys-DA micelles, which was basically consistent with previous work by Vakilzadeh et al. [21].

### 2.5. Dox-Encapsulation and Characterization of LC-Cys-OA/LC-Cys-DA/LC-Cys-OA Micelles

**Figure 4:**
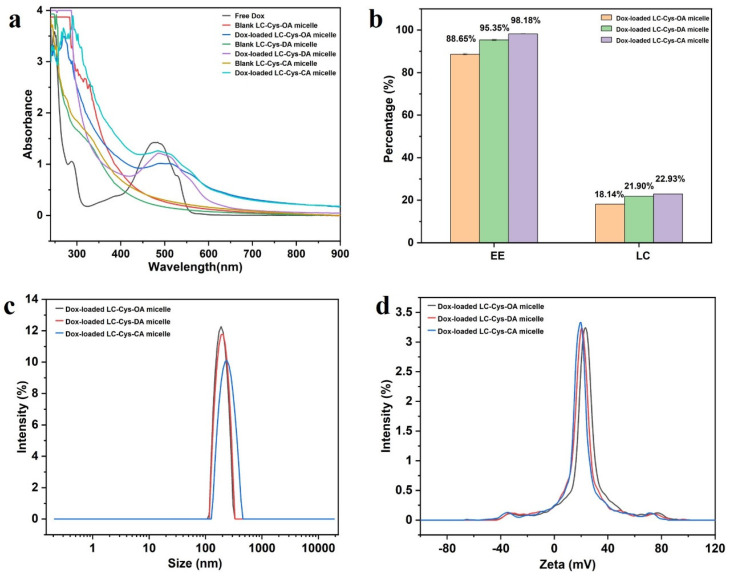
The UV–is spectra of free Dox, three blank micelles and three Dox-loaded micelles (**a**). The EE and LC of Dox in LC-Cys-OA, LC-Cys-DA and LC-Cys-CA micelles (**b**). The size distribution (**c**) and zeta potential (**d**) of three Dox-loaded micelles.

In a view of Figure 4a, a characteristic peak of Dox at 484 nm was found in free Dox and three Dox-loaded micelle solutions, while three blank micelles had no obvious absorption peak at the range of 450–900 nm, which verified successful encapsulation of Dox into the core of the three blank micelles [22].

Encapsulation efficiency is an important property, which could directly affect the clinical application of Dox-loaded micelles [23]. As presented in Figure 4b, with the increase of hydrophobic chain length, the DEE of Dox in the LC-Cys-OA, LC-Cys-DA and LC-Cys-CA micelles increased from 88.65% to 98.18% along with an increased DLC from 18.14% to 22.93%. This finding is in accordance with literature data [24]. Analysis of particle sizes of the three Dox-loaded micelles found that the gradually increased average particle size from 202.86 nm to 235.35 nm was accompanied as the chain length of hydrophobic groups increased from eight to sixteen carbons (Figure 4c). A possible reason was that the LC-Cys-CA micelle with longer hydrophobic chain length had a higher loading capacity for Dox compared with LC-Cys-OA and LC-Cys-DA micelles. Moreover, Figure 4d displays that the zeta potential of the three Dox-loaded micelles remained basically unchanged or slightly decreased with the increase of hydrophobic chain lengths, which was in agreement with the outcome of the blank micelles.

### 2.6. In Vitro Stimuli-Responsive Release

**Figure 5:**
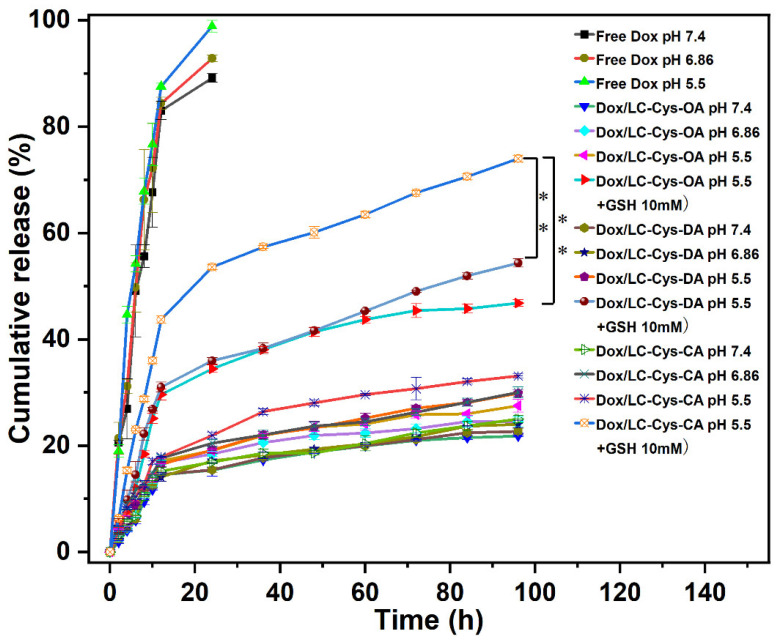
GSH triggered release of Dox from the LC-Cys-OA, LC-Cys-DA and LC-Cys-CA micelles in four different conditions. The results are described as average value ± SD (n = 3, ** *p* < 0.01).

In vitro Dox release behaviors from the three prepared redox-sensitive micelles were examined in four different conditions [25]. As illustrated in Figure 5, a burst release behavior was observed in free Dox, and nearly 100% of Dox was released within 24 h. which was another explanation for the severe toxic effects of Dox. Compared with free Dox, the three Dox-loaded micelles possessed steady and sustained release behavior, and less than 25% of Dox was released from micelles with 96 h in the pH 7.4 condition, which proved that the three Dox-loaded micelles were considerably stable and could prevent drug leakage before reaching the tumor site. Once Dox-loaded micelles were exposed to the pH 5.5 condition, the amount of Dox cumulative release was gradually raised, and approximately 27.51%, 29.85% and 33.10% Dox released from the LC-Cys-OA, LC-Cys-DA and LC-Cys-CA micelles, which might be due to the improved solubility of Dox depending on protonation of the amine groups [26]. After adding 10 mM GSH, which mimics the tumor microenvironment, the release percentage of Dox was promoted significantly, and nearly 46.80%, 54.35% and 73.97% encapsulated Dox was released from the LC-Cys-OA, LC-Cys-DA and LC-Cys-CA micelles after 96 h, which was 2 times higher than that in the pH 5.5 condition without GSH. These results showed that the three micelles containing disulfide bonds were sensitive to the redox-responsive environment, which was a major advantage of cancer treatment [27]. Moreover, the release of Dox from the three prepared micelles presented an increased trend with an increasing of hydrophobic chain lengths, which was related to the redox sensitivity and encapsulation capacity of the three Dox-loaded micelles. Taken together, the LC-Cys-CA micelle was an ideal nanocarrier, which could maintain stability in normal blood but achieved accelerated release in cancer cells.

**Figure 6:**
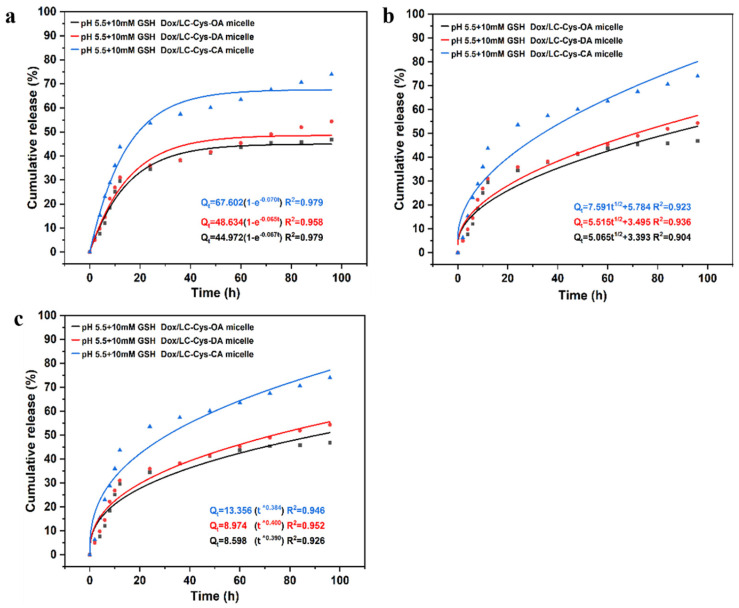
Dox release fitting curves from three drug-loaded micelles by first-order kinetics (**a**), Higuchi kinetics (**b**) and the Ritger–Peppas model (**c**).

For evaluating Dox release mechanisms from three micelles with different hydrophobic chain lengths, as given in Figure 6, a first-order model was selected as the most appropriate model, which displayed the highest correlation with the first-order model (R^2^ = 0.98, 0.96 and 0.98). It was also an indication that Dox release of the three micelles was a positive correlative with a concentration difference inside and outside of the dialysis tube. These results were similar to curcumin release from Pluronic mixed micelle [28].

### 2.7. In Vitro Antitumor Activity of Three Dox-Loaded Micelles

**Figure 7:**
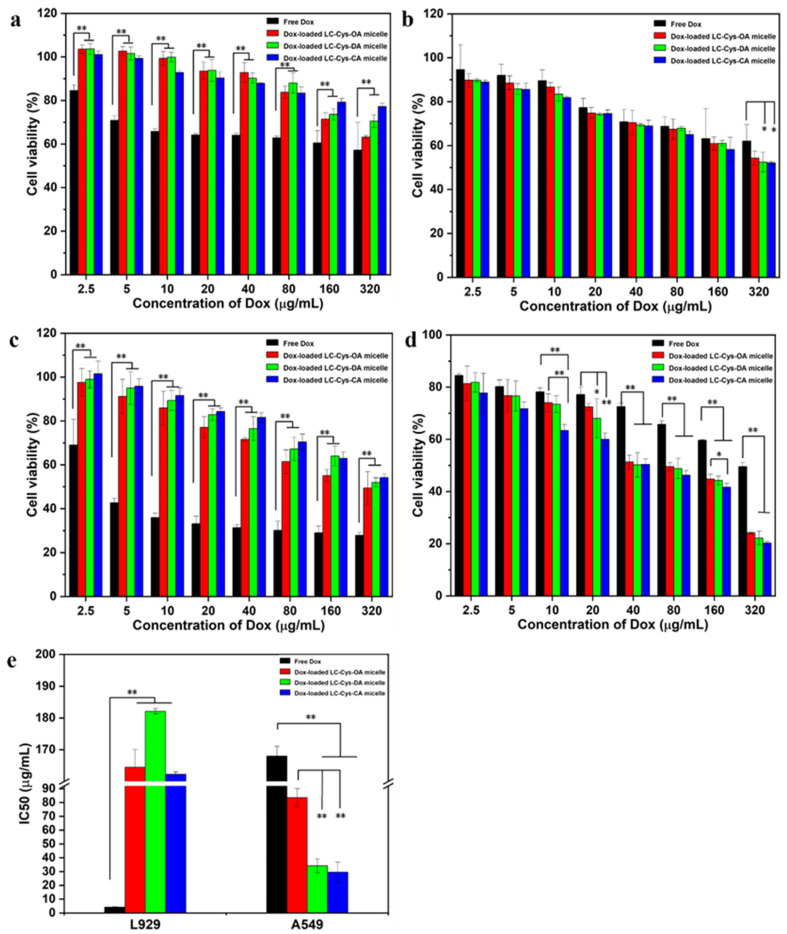
MTT assay of L929 cell (**a**,**c**) and A549 cell (**b**,**d**) treated with free Dox and the three Dox-loaded micelles for 24 h and 48 h. The IC50 values of free Dox and the three Dox-loaded micelles against L929 cells and A549 cells for 48 h incubation (**e**). The results are described as average value ± SD (n = 3, * *p* < 0.05 and ** *p* < 0.01).

The results are depicted in Figure 7. Among all samples, free Dox possessed the highest potent cytotoxicity against L929 cells. When the concentration of Dox was elevated up to 320 μg/mL, the cell viability of free Dox was nearly 90%, 81% and 74% of the three Dox-loaded micelles for 24 h (Figure 7a). As the incubation time extended to 48 h, an obviously reduced cell survival rate was found in the group dealt with the three Dox-loaded micelles (Figure 7c). Meanwhile, Figure 7e shows that free Dox had a IC50 value of 4.25 μg/mL, which was significantly lower than that of the three Dox-loaded micelles. Moreover, Figure S3 shows that the number of living cells in images of L929 cell dealt with the three Dox-loaded micelles (320 μg/mL) were more than that of free Dox. The possible reason was that low GSH concentration of L929 cells might not be sufficient to break disulfide bonds and encapsulated Dox could hardly be released from the micelles, leading to weaker cytotoxicity than free Dox [29].

For A549 cells, the three Dox-loaded micelles were more effective in killing cancer cells compared with free Dox. As given in Figure 7b,d, more than 62% and 49% cell viabilities of A549 cells were treated with 320 μg/mL of free Dox for 24 h and 48 h. In comparison, nearly 54%, 52%, 52% and 24%, 22%, 20% survival ratio of A549 cells dealt with the three Dox-loaded micelles for 24 h and 48 h, respectively. Meanwhile, Figure 7e shows that the three Dox-loaded micelles had IC50 values of 83.51 μg/mL, 34.17 μg/mL and 29.49 μg/mL against A549 cells while the IC50 value of Dox was 167.99 μg/mL. It suggested that the three Dox-loaded micelles had better anti-tumor activity than free Dox. The result might be attributed to the better solubility of Dox in micelles and the enhanced internalization of Dox-loaded micelles in tumor cells [30,31]. In addition, studies found that Dox-loaded LC-Cys-CA micelles exhibited the highest cytotoxicity among all samples, which could be explained by the strong redox-sensitive behavior and the loading ability [32]. The result was consistent with the data of stimuli-responsive release assays.

### 2.8. In Vitro Cellular Uptake

**Figure 8:**
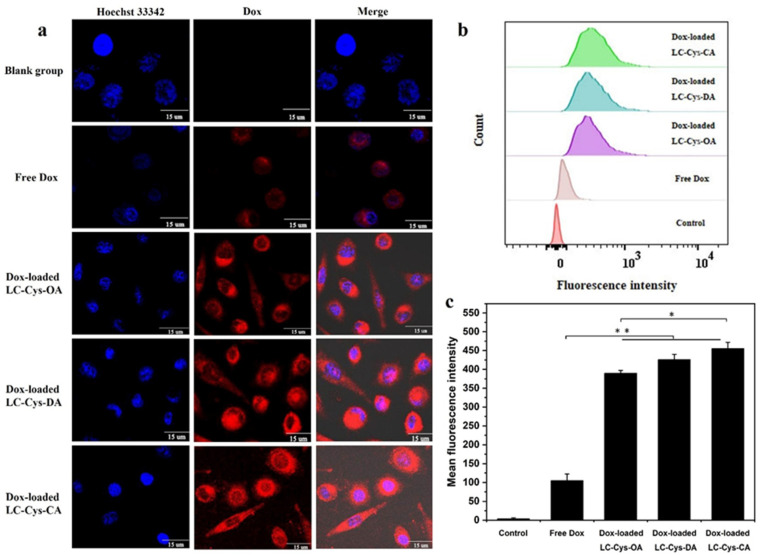
CLSM images (**a**), FCM analyses (**b**) and mean fluorescence intensity (**c**) of A549 cells dealt with free Dox and the three Dox-loaded micelles (50 μg/mL) for 4 h. Nucleus was stained by Hoechst 33342 (blue). Dox showed red fluorescence. Scale bars: 15 μm. The results are described as average value ± SD (n = 3, * *p* < 0.05 and ** *p* < 0.01).

It was foreseeable that three redox-sensitive Dox-loaded micelles would be disintegrated and rapidly released Dox in tumor cells. To confirm this view, the cellular uptake efficiency of the three Dox-loaded micelles was evaluated by capturing red fluorescence of A549 cells in Figure 8a. Free Dox existed in nuclei whereas the red fluorescence of the three Dox-loaded micelles was distributed mainly in cell cytoplasm. One possible reason for this discrepancy is the difference in physicochemical properties of free Dox and Dox-loaded micelles, especially the difference in particle size [33]. Meanwhile, compared with the free Dox group, enhanced red fluorescence was observed in Dox-loaded micelles, which implied that more Dox-loaded micelles were internalized into A549 cells. It is possible that Dox-loaded micelles with a positive charge exhibited accelerated transport into cells through clathrin-mediated endocytosis, which was similar to a previous report [34]. In addition, it was apparent that Dox-loaded LC-Cys-CA micelles possessed brighter fluorescence than the other two Dox-loaded micelles. This result is consistent with cytotoxicity data.

In addition, the mean fluorescence intensities of all samples were quantitatively analyzed by FCM [35]. Figure 8b reveals that the curve of A549 cells treated with the three redox-sensitive Dox-loaded micelles obviously shifts toward high fluorescence intensity compared with free Dox. Meanwhile, Figure 8c demonstrates that the mean fluorescence intensities of A549 cells dealt with the three redox-sensitive Dox-loaded micelles were 3.70, 4.04 and 4.32 times higher than that of free Dox, which was in agreement with CLSM assay. All of the above results strongly proved that the three prepared Dox-loaded micelles could be transferred efficiently into cancer cells and stimuli-responsive released Dox in the redox environment, which would be a benefit for tumor therapy [36].

### 2.9. Antimigratory Activities of Three Dox-Loaded Micelles

**Figure 9:**
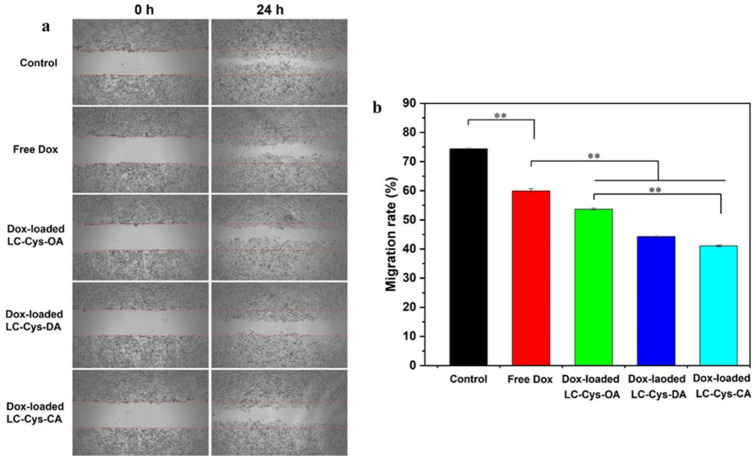
Representative scratch assay image (**a**) and quantification evaluation of migration rate (**b**) of A549 cells dealt with free Dox and the three Dox-loaded micelles for 0 h and 24 h. The results are described as average value ± SD (n = 3, and ** *p* < 0.01).

A scratch assay was carried out to assess antimigratory activities of free Dox and the three Dox-loaded micelles against A549 cells, as presented in Figure 9. A549 cells in the control group exhibited high migration ability, and most of the cells readily migrated to the scratch area, while a modest suppressive migration effect on A549 cell was presented in free Dox, which matches with Zhang et al.’s work [37]. Moreover, the three Dox-loaded micelles demonstrated a notable inhibitory effect on the migration of A549 cells. Interestingly, Dox-loaded LC-Cys-CA micelles hold a stronger inhibitory potency with a migration rate of 41.05% compared with the other two Dox-loaded micelles.

## 3. Materials and Methods

### 3.1. Materials and Reagents

Low molecular weight chitosan (LC) with a 5 kDa molecular weight was bought from Golden-Shell Pharmaceutical Co., Ltd. (Taizhou, China) and used as received. *N, N’*-carbonyldiimidazole (CDI), Cys·2HCl (97%), OA (99%), DA (98%), CA (99%), Dox·HCl (98%), triethylamine (TEA, 98%) and pyrene (97%) were obtained from Macklin Biochemical Co., Ltd. (Shanghai, China). Hoechst 33342, paraformaldehyde (PA, 4%), MTT and GSH were procured from Yuanye Bio-Technology Co., Ltd. (Shanghai, China). Fetal bovine serum (FBS) and Dulbecco’s modified Eagle’s medium (DMEM) were provided by Gibco BRL (Grand Island, NY, USA). Normal mouse fibroblasts cells (L929) and human lung cancer cells (A549) were provided by the Cell Bank of the Chinese Academy of Science (Shanghai, China).

### 3.2. Synthesis of LC-Cys Conjugate

The LC-Cys conjugate was prepared according to a previously published report with slight modifications [38]. Succinctly, LC (3.22 g, 20 mM) was dissolved in DMSO solution (40 mL) and reactivated by CDI at a molar ratio of 1:1 for 12 h under a nitrogen atmosphere. Meanwhile, Cys·2HCl (40 mM, 9.29 g) was dissolved completely in DMSO solution (30 mL) under a vigorous stirring condition. Afterwards, the LC/DMSO solution was slowly added into the Cys/DMSO solution within 12 h. The mixture solutions were continued to react at 60 °C under the N2 atmosphere. After 24 h, the mixture was poured into a beaker containing an ethanol solution (400 mL) followed by filtration to obtain a crude product. Then the product was dissolved again in DMSO and precipitated with ethanol solution three times. Finally, the purified product of LC-Cys conjugate was collected by lyophilization.

### 3.3. Synthesis of Amphiphilic LC-Cys-OA/DA/CA Conjugates

The amphiphilic LC-Cys-OA, LC-Cys-DA and LC-Cys-CA conjugates were synthesized as reported in Sun’s work [39]. Briefly, the amino groups of LC-Cys conjugate (2.03 g) were activated by CDI at a molar ratio of 1:1. After 12 h, OA (6 mM, 0.78 g), DA (6 mM, 1.14 g) and CA (6 mM, 1.61 g) were completely dissolved into 6 mL of N, N-dimethylformamide at 60 °C, respectively, and added dropwise to the above reaction system to continue to react for another 24 h at 60 °C. Afterwards, 200 mL of acetone solution was added and the precipitated solid was separated by filtration. Finally, the products (LC-Cys-OA, LC-Cys-DA and LC-Cys-CA) were obtained by lyophilization.

### 3.4. Characterization of Conjugates

The FTIR spectra of LC and three amphiphilic conjugates (LC-Cys-OA/DA/CA) were tested on a Nicolet iS50 spectrometer (Thermo, Waltham, MA, USA). The samples were mixed with KBr and grinded to powder followed by detection in the region of 4000–400 cm^−1^. The ^1^H NMR spectras of all samples were acquired on a Bruker AVIII-500 Spectrometer. The amphiphilic LC-Cys-OA, LC-Cys-DA and LC-Cys-CA conjugates were dissolved in the mixed solution of D_2_O/DMSO-*d*6 (1:10, *v*/*v*), whereas LC was dissolved in D_2_O. The DS of all samples was quantitatively evaluated by ^1^H NMR using the integral function in MestReNova [40]. For instance, the DS of samples was described by the following formula:

$${\mathrm{DS}}_{1}=\frac{5}{4\;\times \;(\int H_{1}/\int H_{2}-1)}$$

$${\mathrm{DS}}_{2}=\frac{(\int H_{3}/\int H_{4})\;\times \;\mathrm{DS}1\;\times \;5}{3}$$

where DS_1_ was the DS of LC-Cys; DS_2_ was the DS of LC-Cys-OA, LC-Cys-DA, LC-Cys-CA; ∫H1 was the integral areas of hydrogen at 3.20–3.98 ppm of LC-Cy derivate; ∫H2 was the integral areas of hydrogen at 2.56–3.05 ppm of LC-Cys derivate; ∫H3 and ∫H4 were the integral areas of hydrogen at 2.56–3.05 ppm and 0.84 ppm of LC-Cys-OA, LC-Cys-DA, LC-Cys-CA derivates.

### 3.5. Preparation of Blank Micelle and Dox Loaded Micelle

#### 3.5.1. Preparation of Blank Micelle

The blank micelles of LC-Cys-OA, LC-Cys-DA and LC-Cys-CA were prepared by a simple ultrasound-dialysis method [41]. In brief, 10 mg of conjugates was dissolved completely in 1 mL of DMSO solution and then 1 mL water was added under vigorous stirring. The mixture solutions were dialyzed against water (MWCO = 100 Da) for 4 h. Subsequently, the resultant solutions were sonicated for 30 min (2 s on, 1 s off) by probe sonicator (JY92-ⅡDN, Ningbo, China) at 135 W in an ice condition to obtain three self-assembled micelles.

#### 3.5.2. Preparation of Dox Loaded Micelles

The procedure of Dox-loaded micelles was similar to that of blank micelles. Firstly, Dox·HCl was mixed with triethylamine at a molar ratio of 3:1 to obtain free Dox. After 48 h, Dox solution (67 µL, 15 mg/mL) was slowly dropped into LC-Cys-OA, LC-Cys-DA or LC-Cys-CA solutions with gentle stirring and sonicated for 25 min to ensure the effective encapsulation of Dox into the LC-Cys-OA, LC-Cys-DA and LC-Cys micelle solutions. In addition, the Dox loading efficiency (DEE) and Dox loading content (DLC) in LC-Cys-OA, LC-Cys-DA and LC-Cys-CA micelles were estimated by determining the absorbance of Dox at 484 nm using UV spectrophotometry. The special equations are as follows:

$$\mathrm{DEE}\;(\%)=\frac{\mathrm{Amount}\;\mathrm{of}\;\mathrm{loaded}\;\mathrm{Dox}}{\mathrm{Amount}\;\mathrm{of}\;\mathrm{initial}\;\mathrm{Dox}}\;\times \;100$$

$$\mathrm{DLC}\;(\%)=\frac{\mathrm{Amount}\;\mathrm{of}\;\mathrm{loaded}\;\mathrm{Dox}}{\mathrm{Total}\;\mathrm{amount}\;\mathrm{of}\;\mathrm{Dox}\;\mathrm{loaded}\;\mathrm{micelle}}\;\times \;100$$

### 3.6. Characterization of Blank Micelles and Dox Loaded Micelles

The hydrophobic pyrene was used to determine the CMC values of the three blank micelles as previously reported [42]. Briefly, a Pyrene/acetone solution (0.25 mL) was added to a series of brown test tubes. After acetone evaporation, 2.25 mL of LC-Cys-OA, LC-Cys-DA or LC-Cys-CA micelles with different concentrations from 0.005 to 0.4 mg/mL was added to each test tube to obtain a 3 × 10^−5^ M final pyrene concentration. The fluorescence spectra of all samples was monitored by a fluorescence spectrophotometer (Hitachi-F-7000, Tokyo, Japan) at an emission wavelength of 333 nm. The CMC value was determined as the cross-point of two straight lines by drawing the intensity ratio of (I_373_/I_383_) as a function of logarithm micelle solution concentrations.

The particle size and zeta potential of the three blank micelles and Dox-loaded micelles were analyzed using a Litesizer 500 dynamic light scattering (DLS) (Austria). The morphologies of the three blank micelles were characterized by JEM-1200EX transmission electron microscopy (Japan). The storage stability of the blank three micelles was evaluated by observing the changes of particle size, polydispersity index and zeta potential within eight days.

### 3.7. Stimuli-Responsiveness Behavior of Blank Micelles Triggered by GSH

The stimuli-responsiveness behavior of the three blank LC-Cys-OA, LC-Cys-DA and LC-Cys-CA micelle solutions in response to a 10 mM GSH condition was monitored by DLS method. Typically, the three blank micelle solutions and GSH (92.10 mg) were successively added into a round-bottomed flask. Then the mixed solutions were mildly stirred at 37 °C at a speed of 200 rpm. At predetermined time points (0, 6, 12 and 24 h), the size distribution and mean particle size were determined by DLS method to assess the redox-response behavior of the three blank micelles.

### 3.8. In Vitro Biosafety Evaluation

#### 3.8.1. Hemolysis Assay

The blood compatibility of the three redox-sensitive micelles was estimated by hemolysis assay. Succinctly, the obtained red blood cells (RBCs) were centrifuged at 4000 rpm for 5 min followed by resuspending with saline to obtain 2% suspension. Then equal volumes of RBCs suspensions (2%) were mixed with blank LC-Cys-OA, LC-Cys-DA or LC-Cys-CA micelles at the range of 0.5–2.0 mg/mL and incubated for 1 h in a thermostatic water bath at 37 °C. In parallel, a NaCl solution (0.9%) and deionized water were regarded as negative and positive control groups, which were treated with the same condition as the samples. Finally, all samples were measured using a UV-vis spectrometer (PerkinElmer, Waltham, MA, USA) at 540 nm. The hemolysis rate was calculated using the following equation:

$$\mathrm{Hemolysis}\;\mathrm{rate}\;(\%)=\frac{\left({\mathrm{OD}}_{\mathrm{sample}}\;-\;{\mathrm{OD}}_{\mathrm{negative}}\right)}{\left({\mathrm{OD}}_{\mathrm{positive}}\;-\;{\mathrm{OD}}_{\mathrm{negative}}\right)}\;\times \;100$$

where OD_sample_, OD_negative_ and OD_positive_ show the absorbance of 2% RBCs suspensions treated with all samples, NaCl solution (0.9%) and deionized water, respectively.

#### 3.8.2. Cytotoxicity Assay

L929 cells and A549 cells were seeded into 96-well plates (8 × 10^3^ cells/well). After 36 h, blank micelle solution with various concentrations (10–1000 μg/mL) was added and incubated for 48 h. Afterward, all sample solutions were removed and stained by MTT solution (100 μL, 0.5 mg/mL) for 4 h. Later, 150 μL of DMSO solution was added to completely dissolve the purple formazan crystals. Finally, a microplate reader (SuperMax, Shanghai, China) was used to test the absorbance of all samples at a wavelength of 490 nm and cell survival rate was calculated by the following equation:

$$\mathrm{Cell}\;\mathrm{survival}\;\mathrm{rate}\;(\%)=\frac{A_{\mathrm{sample}}-A_{\mathrm{blank}}}{A_{\mathrm{control}}-A_{\mathrm{blank}}}\;\times \;100$$

where A_sample_ and A_control_ were absorbance of cells incubated with the three blank micelles and culture medium, while A_blank_ meant the absorbance of culture medium without cells.

### 3.9. Redox Responsive Drug Release and Release Mechanism

#### 3.9.1. In Vitro Drug Release Behavior

The dialysis method was used to explore the Dox release behavior from LC-Cys-OA, LC-Cys-DA and LC-Cys-CA micelles in response to different conditions [43]. The PBS solution (pH = 7.4) and PBS solution (pH = 5.5) containing 10 mM GSH were selected to mimic a normal physiological environment and tumor intracellular environment. Briefly, 5 mL of free Dox solution and Dox-loaded micellar solutions were placed in dialysis bags (MWCO: 8000–12,000 Da), which were dialyzed against 150 mL of different PBS solutions with a speed of 100 rpm at 37 °C. At the scheduled time, 3 mL of dialysate was collected and replenished with an equivalent volume of freshly prepared PBS solution. The amount of released Dox in the dialysis bags was quantified by UV–vis spectrophotometer (PerkinElmer, USA) at 484 nm. The cumulative release (%) of Dox was assessed using the following equation:

$$\mathrm{Cumulative}\;\mathrm{Dox}\;\mathrm{release}\;(\%)=\frac{M_{i}}{M_{\mathrm{total}}}\;\times \;100$$

where M_i_ and M_total_ represent the total release Dox from micelle at time i and the initial Dox loaded in micelle, respectively.

#### 3.9.2. Drug Release Mechanism

The release data were fitted to three kinetic patterns (first-order kinetic models, Higuchi model and Ritger–Peppas model) to find the most suitable kinetics model and further explore the in vitro drug release mechanism [44,45].

Equation (1) was a first-order model that drug release was related to drug concentration loaded in micelles:

M_t_ = M_0_ (1 − e^−k1t^) (1)

Equation (2) was a Higuchi model that was associated with a drug release rate from matrix:

(2) $$M_{t}=k_{H}\sqrt{t}$$

Equation (3) was a Ritger–Peppas model that defined the drug delivery mode from the carrier system:

M_t_ = k_R_t^n^ (3)

where M_t_ and M_0_ meant the cumulative Dox release amount from micelle at time t and initial time; k_1_, k_H_ and k_R_ were release constants, which were closely related to structure and characteristics of different micelles; n represented the diffusional exponent that referred to the transport mechanism of Dox from micelles.

### 3.10. In Vitro Antitumor Activity Assay

The antitumor activity of free Dox and the three Dox-loaded micelles was evaluated by the MTT method as described by Guo et al. [46]. The specific procedure is similar to cytotoxicity assay (3.8.2). L929 cells and A549 cells were seeded into a 96-well plate. Free Dox and the three Dox-loaded micelle solutions with different concentrations (2.5–320 μg/mL) were added to incubate for 24 h and 48 h, respectively. Subsequently, a MTT solution (0.5 mg/mL,100 μL) was dropped into each well and tested at 490 nm by a microplate reader.

### 3.11. In Vitro Cellular Uptake

CLSM and FCM techniques were carried out to test whether free Dox and the three Dox-loaded micelles could be internalized into cancer cells [47]. For fluorescence microscopy observation, A549 cells were seeded in a confocal dish (1.5 × 10^5^ cells/dish). The original medium was replaced by free Dox and the three Dox-loaded micelles solutions (50 μg/mL) and continued to incubate for 4 h. Afterward, A549 cells were stained with 5 μg/mL of Hoechst 33342 solution for 20 min and observed by CLSM (FluoView FV1000, Olympus, Tokyo, Japan). For flow cytometry analysis, A549 cells were washed, digested, collected and detected by FCM (BD FACSAria II) to quantitatively measure fluorescent intensity.

### 3.12. Wound Healing Assay

The wound healing assay was operated to evaluate the migration inhibition efficacy of free Dox and the three Dox-loaded micelles in A549 cells [48]. Succinctly, A549 cells were plated in 6-well dishes at a density of 5 × 10^5^ cells/dish and a linear scratch wound was created by a 200 μL sterile pipette tip. After that, free Dox and the three Dox-loaded micelles were added, the images of all samples were collected by inverted microscope (Olympus, Tokyo, Japan) and the migration percentage of wound was calculated using the following equation:

$$\mathrm{Migration}\;\mathrm{percentage}\;(\%)=\frac{\left(\mathrm{migration}\;\mathrm{distance}\right)}{\left(\mathrm{initial}\;\mathrm{scratch}\;\mathrm{width}\right)}\;\times \;100$$

### 3.13. Statistical Analysis

All quantitative data were expressed as average values ± SD from at least triplicate measurements. One-way ANOVA in SPSS 17 software was used to analyze the differences among groups. The differences were considered significant and extremely significant when * *p* < 0.05 and ** *p* < 0.01.

## 4. Conclusions

In this work, we successfully prepared three amphiphilic conjugates with different hydrophobic chain lengths (LC-Cys-OA, LC-Cys-DA and LC-Cys-CA) by the catalytic action of CDI, which could further self-assemble into core-shell structural micelles to maximize the therapeutic efficacy of Dox via regulating the chain length of hydrophobic segments. Three redox-sensitive micelles with a spherical structure had small CMC values of 0.075–0.048 mg/mL, ideal particle size of 147.95–101.47 nm and positive zeta potential of +21.84–+24.26 mV. Meanwhile, three redox-sensitive micelles exhibited excellent stability and desirable biosafety as nanocarriers. The loading capacities of these three micelles were investigated by determining the DEE and DLC values. The result showed that the redox micelle with a longer hydrophobic chain length had higher DEE and DLC values than the other two micelles. The redox sensitivity of the three prepared micelles were verified by observing size distribution change and measuring Dox release in 10 mM GSH mimicking the tumor microenvironment. The result indicated that Dox-loaded LC-Cys-CA micelles showed the strongest redox sensitivity to tumor cells among the three Dox-loaded micelles. In vitro release assay displayed that the three Dox-loaded micelles had accelerated Dox release behavior in the redox environment of tumor cells due to the cleavage of disulfide linkage and dissociation of the whole micelle. Moreover, Dox release from the three redox-responsive micelles followed a first-order model and showed a concentration-dependent release mechanism. Combined with antitumor activity, cellular uptake assay revealed that the three Dox-loaded micelles could rapidly deliver into a tumor and efficiently kill the tumor cell. Particularly, an enhanced transport efficiency and superior antitumor effect were found in Dox-loaded LC-Cys-CA micelles with longer hydrophobic chain length compared with others. In addition, the three Dox-loaded micelles presented considerable suppressive effects on cancer cell migration. In a nutshell, the most significant advantage of designing the three amphiphilic conjugates was that the three prepared redox micelles could provide an effective platform to improve the transport rate of hydrophobic Dox, enhance release efficiency of the wrapped Dox and achieve the best antitumor effect by regulating the chain length of hydrophobic groups.

## Data Availability

All data are contained in the manuscript and Supplementary Material.

## Supplementary Materials

The following supporting information can be downloaded at: https://www.mdpi.com/article/10.3390/md22010018/s1, Figure S1: Synthetic route for amphiphilic LC-Cys-OA/LC-Cys-DA/LC-Cys-CA conjugates (a), FTIR (b) and 1H NMR spectra (c) of LC and amphiphilic conjugates; Figure S2: TEM images of LC-Cys-OA, LC-Cys-DA and LC-Cys-CA micelles; Figure S3: The morphology of L929 cell and A549 cell dealt with free Dox and Dox-loaded LC-Cys-OA, Dox-loaded LC-Cys-DA and Dox-loaded LC-Cys-CA micelles for 24 h (A) and 48 h (B).

## References

[1] Sikder A., Vambhurkar G., Amulya E., Bagasariya D., Famta P., Shah S., Khatri D.K., Singh S.B., Sinha V.R., Srivastava S. (2022). Advancements in redox-sensitive micelles as nanotheranostics: A new horizon in cancer management. J. Control. Release.

[2] Sun Y., Zou W., Bian S., Huang Y., Tan Y., Liang J., Fan Y., Zhang X. (2013). Bioreducible PAA-g-PEG graft micelles with high doxorubicin loading for targeted antitumor effect against mouse breast carcinoma. Biomaterials.

[3] Shymborska Y., Budkowski A., Raczkowsk J., Donchak V., Melnyk Y., Vasiichuk V. (2023). Switching it Up: The Promise of Stimuli-Responsive Polymer Systems in Biomedical Science. Chem. Rec..

[4] Jeong G.W., Jeong Y.I., Nah J.W. (2019). Triggered doxorubicin release using redox-sensitive hyaluronic acid-g-stearic acid micelles for targeted cancer therapy. Carbohydr. Polym..

[5] Liu Y.-S., Huang S.-J., Huang X.-S., Wu Y.-T., Chen H.-Y., Lo Y.-L., Wang L.-F. (2016). The synthesis and comparison of poly(methacrylic acid)–poly(ε-caprolactone) block copolymers with and without symmetrical disulfide linkages in the center for enhanced cellular uptake. RSC Adv..

[6] Birlik Demirel G., Bayrak Ş. (2022). Ultrasound/redox/pH-responsive hybrid nanoparticles for triple-triggered drug delivery. J. Drug Deliv. Sci. Technol..

[7] Lin D., Xiao L., Qin W., Loy D.A., Wu Z., Chen H., Zhang Q. (2022). Preparation, characterization and antioxidant properties of curcumin encapsulated chitosan/lignosulfonate micelles. Carbohydr. Polym..

[8] Itoo A.M., Paul M., Ghosh B., Biswas S. (2022). Oxaliplatin delivery via chitosan/vitamin E conjugate micelles for improved efficacy and MDR-reversal in breast cancer. Carbohydr. Polym..

[9] Shirani S., Varshosaz J., Rostami M., Mirian M. (2022). Redox responsive polymeric micelles of gellan gum/abietic acid for targeted delivery of ribociclib. Int. J. Biol. Macromol..

[10] Liu M., Du H., Zhai G. (2016). Self-assembled nanoparticles based on chondroitin sulfate-deoxycholic acid conjugates for docetaxel delivery: Effect of degree of substitution of deoxycholic acid. Colloids Surf. B Biointerfaces.

[11] Lu A., Petit E., Jelonek K., Orchel A., Kasperczyk J., Wang Y., Su F., Li S. (2020). Self-assembled micelles prepared from bio-based hydroxypropyl methyl cellulose and polylactide amphiphilic block copolymers for anti-tumor drug release. Int. J. Biol. Macromol..

[12] Li M., Zhao Y., Sun J., Chen H., Liu Z., Lin K., Ma P., Zhang W., Zhen Y., Zhang S. (2022). pH/reduction dual-responsive hyaluronic acid-podophyllotoxin prodrug micelles for tumor targeted delivery. Carbohydr. Polym..

[13] Luo Q., Han Q., Chen L., Fan X., Wang Y., Fei Z., Zhang H., Wang Y. (2020). Redox response, antibacterial and drug package capacities of chitosan-alpha-lipoic acid conjugates. Int. J. Biol. Macromol..

[14] Yang H.Y., Jang M.-S., Gao G.H., Lee J.H., Lee D.S. (2016). Construction of redox/pH dual stimuli-responsive PEGylated polymeric micelles for intracellular doxorubicin delivery in liver cancer. Polym. Chem..

[15] Liu M., Du H., Khan A.R., Ji J., Yu A., Zhai G. (2018). Redox/enzyme sensitive chondroitin sulfate-based self-assembled nanoparticles loading docetaxel for the inhibition of metastasis and growth of melanoma. Carbohydr. Polym..

[16] He X.-H., Zhao M., Tian X.-Y., Lu Y.-J., Yang S.-Y., Peng Q.-R., Yang M., Jiang W.-W. (2022). Redox-responsive nano-micelles containing trisulfide bonds to enhance photodynamic efficacy of zinc naphthalocyanine. Chem. Phys. Lett..

[17] Yadav S., Kumar P., Jo S.-H., Park S.-H., Lee W.-K., Yoo S., Lim K.T. (2022). Redox-responsive properties of core-cross-linked micelles of poly(ethylene oxide)-b-poly(furfuryl methacrylate) for anticancer drug delivery application. React. Funct. Polym..

[18] Li J., Liu P. (2022). Facile Synthesis of a Redox-Responsive Hyperbranched Polymer Prodrug as a Unimolecular Micelle for the Tumor-Selective Drug Delivery. Bioconjug. Chem..

[19] Sang X., Yang Q., Shi G., Zhang L., Wang D., Ni C. (2018). Preparation of pH/redox dual responsive polymeric micelles with enhanced stability and drug controlled release. Mater. Sci. Eng. C Mater. Biol. Appl..

[20] Zhang H., Liu X., Xu K., Du B., Zhu C., Li Y. (2020). Biodegradable polyurethane PMeOx-PU(SS)-PMeOx micelles with redox and pH-sensitivity for efficient delivery of doxorubicin. Eur. Polym. J..

[21] Vakilzadeh H., Varshosaz J., Dinari M., Mirian M., Hajhashemi V., Shamaeizadeh N., Sadeghi H.M. (2023). Smart redox-sensitive micelles based on chitosan for dasatinib delivery in suppressing inflammatory diseases. Int. J. Biol. Macromol..

[22] Hao J., Wang J., Pan H., Sang Y., Wang D., Wang Z., Ai J., Lin B., Chen L. (2022). pH-redox responsive polymer-doxorubicin prodrug micelles studied by molecular dynamics, dissipative particle dynamics simulations and experiments. J. Drug Deliv. Sci. Technol..

[23] Wang Y., Khan A., Liu Y., Feng J., Dai L., Wang G., Alam N., Tong L., Ni Y. (2019). Chitosan oligosaccharide-based dual pH responsive nano-micelles for targeted delivery of hydrophobic drugs. Carbohydr. Polym..

[24] Jelonek K., Li S., Wu X., Kasperczyk J., Marcinkowski A. (2015). Self-assembled filomicelles prepared from polylactide/poly(ethylene glycol) block copolymers for anticancer drug delivery. Int. J. Pharm..

[25] Wang J., Yang G., Guo X., Tang Z., Zhong Z., Zhou S. (2014). Redox-responsive polyanhydride micelles for cancer therapy. Biomaterials.

[26] Gebrie H.T., Addisu K.D., Darge H.F., Birhan Y.S., Thankachan D., Tsai H.C., Wu S.Y. (2022). pH/redox-responsive core cross-linked based prodrug micelle for enhancing micellar stability and controlling delivery of chemo drugs: An effective combination drug delivery platform for cancer therapy. Biomater. Adv..

[27] Han L., Hu L., Liu F., Wang X., Huang X., Liu B., Feng F., Liu W., Qu W. (2019). Redox-sensitive micelles for targeted intracellular delivery and combination chemotherapy of paclitaxel and all-trans-retinoid acid. Asian J. Pharm. Sci..

[28] Kaur J., Singla P., Kaur I. (2022). Labrasol mediated enhanced solubilization of natural hydrophobic drugs in Pluronic micelles: Physicochemical and in vitro release studies. J. Mol. Liq..

[29] Zhao D., Wu J., Li C., Zhang H., Li Z., Luan Y. (2017). Precise ratiometric loading of PTX and DOX based on redox-sensitive mixed micelles for cancer therapy. Colloids Surf. B Biointerfaces.

[30] Jin R., Liu Z., Liu T., Yuan P., Bai Y., Chen X. (2021). Redox-responsive micelles integrating catalytic nanomedicine and selective chemotherapy for effective tumor treatment. Chin. Chem. Lett..

[31] Luo T., Han J., Zhao F., Pan X., Tian B., Ding X., Zhang J. (2019). Redox-sensitive micelles based on retinoic acid modified chitosan conjugate for intracellular drug delivery and smart drug release in cancer therapy. Carbohydr. Polym..

[32] Chiang Y.T., Yen Y.W., Lo C.L. (2015). Reactive oxygen species and glutathione dual redox-responsive micelles for selective cytotoxicity of cancer. Biomaterials.

[33] Wang L., Zhang J., Song M., Tian B., Li K., Liang Y., Han J., Wu Z. (2017). A shell-crosslinked polymeric micelle system for pH/redox dual stimuli-triggered DOX on-demand release and enhanced antitumor activity. Colloids Surf. B Biointerfaces.

[34] Shi C., Guo X., Qu Q., Tang Z., Wang Y., Zhou S. (2014). Actively targeted delivery of anticancer drug to tumor cells by redox-responsive star-shaped micelles. Biomaterials.

[35] Li C., Guan H., Li Z., Wang F., Wu J., Zhang B. (2020). Study on different particle sizes of DOX-loaded mixed micelles for cancer therapy. Colloids Surf. B Biointerfaces.

[36] Li Y., Hu D., Pan M., Qu Y., Chu B., Liao J., Zhou X., Liu Q., Cheng S., Chen Y. (2022). Near-infrared light and redox dual-activatable nanosystems for synergistically cascaded cancer phototherapy with reduced skin photosensitization. Biomaterials.

[37] Zhang T., Liu H., Li Y., Li C., Wan G., Chen B., Li C., Wang Y. (2019). A pH-sensitive nanotherapeutic system based on a marine sulfated polysaccharide for the treatment of metastatic breast cancer through combining chemotherapy and COX-2 inhibition. Acta Biomater..

[38] Zhou L., Liang Q., Li Y., Cao Y., Li J., Yang J., Liu J., Bi J., Liu Y. (2022). Collagenase-I decorated co-delivery micelles potentiate extracellular matrix degradation and hepatic stellate cell targeting for liver fibrosis therapy. Acta Biomater..

[39] Sun C., Li X., Du X., Wang T. (2018). Redox-responsive micelles for triggered drug delivery and effective laryngopharyngeal cancer therapy. Int. J. Biol. Macromol..

[40] Wu C., Yang J., Xu X., Gao C., Lü S., Liu M. (2016). Redox-responsive core-cross-linked mPEGylated starch micelles as nanocarriers for intracellular anticancer drug release. Eur. Polym. J..

[41] Chai Z., Teng C., Yang L., Ren L., Yuan Z., Xu S., Cheng M., Wang Y., Yan Z., Qin C. (2020). Doxorubicin delivered by redox-responsive Hyaluronic Acid-Ibuprofen prodrug micelles for treatment of metastatic breast cancer. Carbohydr. Polym..

[42] Li H., Yang H., Xu J., Gao Z., Wu J., Zhu L., Zhan X. (2022). Novel amphiphilic carboxymethyl curdlan-based pH responsive micelles for curcumin delivery. LWT.

[43] Yang F., Wei P., Yang M., Chen W., Zhao B., Li W., Wang J., Qiu L., Chen J. (2022). Redox-sensitive hyaluronic acid-ferrocene micelles delivering doxorubicin for enhanced tumor treatment by synergistic chemo/chemodynamic therepay. J. Drug Deliv. Sci. Technol..

[44] Mahani M., Bahmanpouri M., Khakbaz F., Divsar F. (2023). Doxorubicin-loaded polymeric micelles decorated with nitrogen-doped carbon dots for targeted breast cancer therapy. J. Drug Deliv. Sci. Technol..

[45] Unagolla J.M., Jayasuriya A.C. (2018). Drug transport mechanisms and in vitro release kinetics of vancomycin encapsulated chitosan-alginate polyelectrolyte microparticles as a controlled drug delivery system. Eur. J. Pharm. Sci..

[46] Guo Z., Liang E., Sui J., Ma M., Yang L., Wang J., Hu J., Sun Y., Fan Y. (2020). Lapatinib-loaded acidity-triggered charge switchable polycarbonate-doxorubicin conjugate micelles for synergistic breast cancer chemotherapy. Acta Biomater..

[47] Qiu X., Ma S., Wang D., Fan Z., Qiu P., Wang S., Li C. (2023). The development of multifunctional sulfated polyguluronic acid-based polymeric micelles for anticancer drug delivery. Carbohydr. Polym..

[48] Nezhadi S., Norouzi P., Rasouli A., Akbari Javar H., Ostad S.N., Dorkoosh F. (2023). Co-delivery of paclitaxel and regorafenib by F127/TPGS mixed micelles for triple negative breast cancer treatment. J. Drug Deliv. Sci. Technol..

