# Supplementary material for: Effect of Hydrophobic Chain Length in Amphiphilic Chitosan Conjugates on Intracellular Drug Delivery and Smart Drug Release of Redox-Responsive Micelle
Source: Mar Drugs. 2023 Dec 27;22(1):18. doi: 10.3390/md22010018 (PMC10821436; doi:10.3390/md22010018)
Supplement: Supplementary file 1 [file marinedrugs-22-00018-s001.zip › marinedrugs-2796612-supplementary.pdf]

Supplementary Material

Effect of hydrophobic chain length in amphiphilic chitosan conjugates on intracellular drug delivery and smart drug release of redox-responsive micelle

Yuting Yuan [1, 2, 3], Wenqiang Tan [1, 3, *], Yingqi Mi [1, 3], Linqing Wang [1, 2, 3], Zhen Qi [4], Zhanyong Guo [1, 2, 3, *]

[1] Key Laboratory of Coastal Biology and Bioresource Utilization, Yantai Institute of Coastal Zone Research, Chinese Academy of Sciences, Yantai 264003, China; ytyuan@yic.ac.cn (Y.Y.); yqmi@yic.ac.cn (Y.M.); linqingwang@yic.ac.cn (L.W.);

[2] University of Chinese Academy of Sciences, Beijing 100049, China

[3] Center for Ocean Mega-Science, Chinese Academy of Sciences, Qingdao 266071, China

[4] College of Life Sciences, Yantai University, Yantai 264005, China; qzqq2022@163.com (Z.Q.);

* Correspondence: zhanyongguo@hotmail.com (Z.G.); wqtan@yic.ac.cn (W.T.); Tel.: +86-535-2109171 (Z.G.); Fax: +86-535-2109000 (Z.G.)

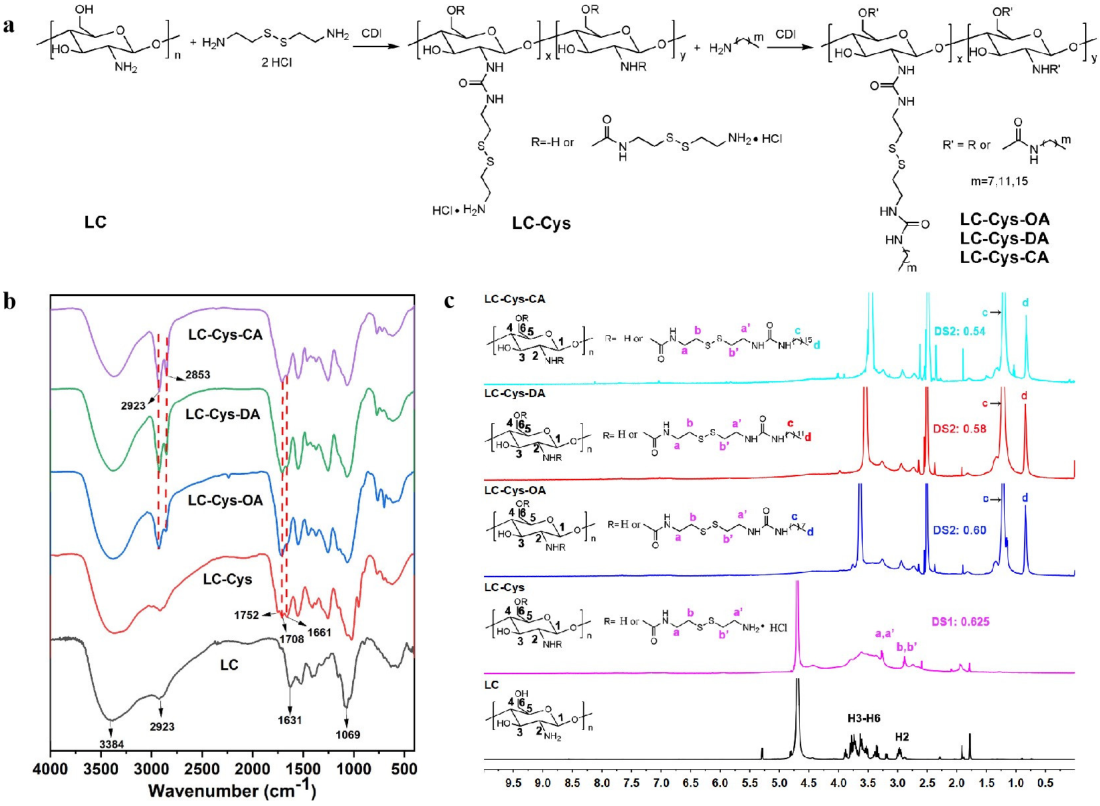

Figure. S1. Synthetic route for amphiphilic LC-Cys-OA / LC-Cys-DA / LC-Cys-CA conjugates (a), FTIR (b) and ^{1}H NMR spectra (c) of LC and amphiphilic conjugates.

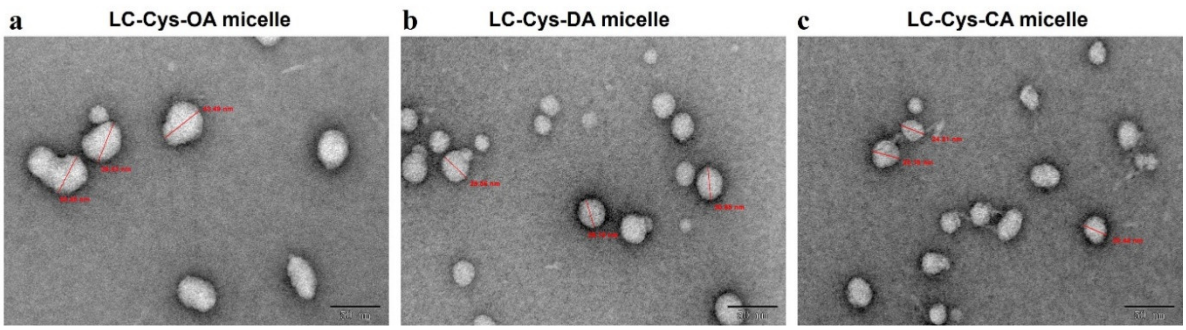

Figure. S2. TEM images of LC-Cys-OA, LC-Cys-DA and LC-Cys-CA micelles.

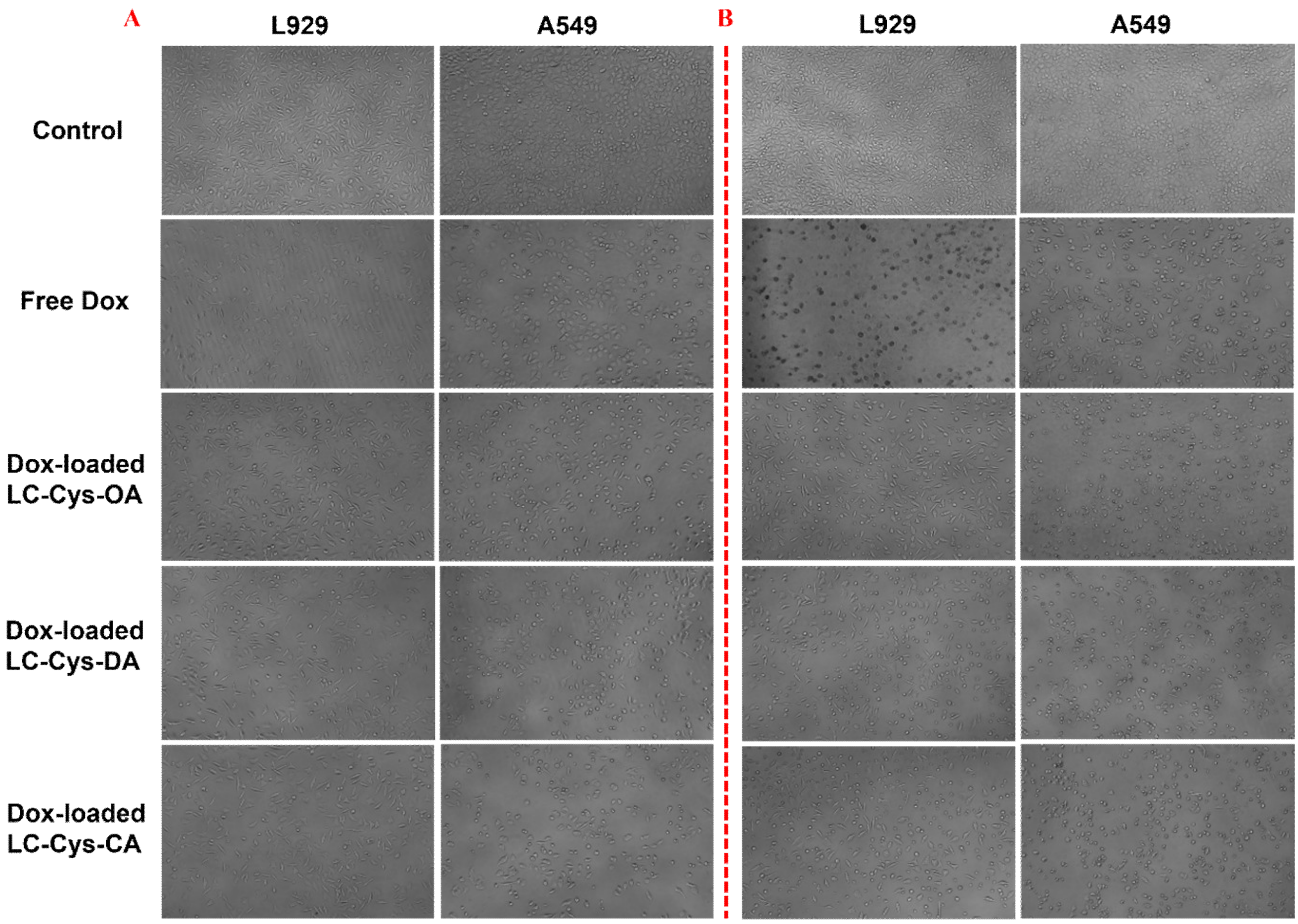

Figure. S3. The morphology of L929 cell and A549 cell dealt with free Dox and Dox-loaded LC-Cys-OA, Dox-loaded LC-Cys-DA and Dox-loaded LC-Cys-CA micelles for 24 h (A) and 48 h (B).
